# Models of good practice to enhance infectious disease care cascades among people who inject drugs: a qualitative study of interventions implemented in European settings

**DOI:** 10.1186/s12913-023-10412-y

**Published:** 2023-12-04

**Authors:** Ilonka Horváth, Otilia Mårdh, Tanja Schwarz

**Affiliations:** 1 Austrian National Public Health Institute (Gesundheit Österreich GmbH, GOEG), Vienna, Austria; 2 European Centre for Disease Prevention and Control (ECDC), Solna, Sweden

**Keywords:** Good practice, PWID, Care continuum, HCV, HIV, Europe

## Abstract

**Background:**

People who inject drugs (PWID) in Europe are at an increased risk of HIV/AIDS, chronic viral hepatitis B (HBV) and C (HCV), and tuberculosis (TB). We aimed to complement the evidence base on interventions optimising their care cascade with evidence from models of good practice (MoGPs) implemented in the EU/EEA and countries from the Eastern European region.

**Methods:**

A model of good practice (MoGP) was defined as (a package of) interventions with proven effectiveness in certain settings that are likely to be replicable and sustainable in other settings or countries. Fifteen MoGPs, identified by the European Centre for Disease Prevention and Control (ECDC) and the European Monitoring Centre on Drugs and Drug Addiction (EMCDDA) following a call launched in 2020, have been analysed. For the 15 MoGPs, a qualitative content analysis was conducted of (i) intervention characteristics and (ii) enabling factors. Information was extracted and summarised for community-based testing, linkage to care and adherence to treatment.

**Results:**

MoGPs emerged from projects implemented in Belarus, Norway, Portugal, the Republic of Moldova, Spain, and the UK alongside the multi-country HepCare project (Ireland, Romania, Spain, the UK) targeting either HCV (6/15) or HIV/AIDS (4/15), alone or combined with HBV, and/or TB (5/15). All MoGPs used packages of interventions, with decentralisation of services (15/15), cooperation among service providers (14/15), integrated services (10/15), peer interventions (12/15), and case management (4/15) reported across all stages of the care cascade. The synthesis of enablers shows that when replicating interventions in other settings, consideration should be given to national (legal) frameworks, characteristics of and proximity between healthcare and service providers, and establishing relations of trust with PWID.

**Conclusion:**

To improve the cascade of care for PWID in European settings, care structures and pathways should be simplified, based on cooperation and multidisciplinary. MoGPs can provide implementation-based evidence on interventions alongside evidence from peer-reviewed literature to optimise the care cascade among PWID.

**Supplementary Information:**

The online version contains supplementary material available at 10.1186/s12913-023-10412-y.

## Background

People who inject drugs (PWID) are still at increased risk of infections such as the human immunodeficiency virus/acquired immunodeficiency syndrome (HIV/AIDS), tuberculosis (TB) and viral hepatitis in the European Union/European Economic Area (EU/EEA) and countries which are part of the Eastern European Neighbourhood Policy (ENP) [1]. Injecting drug use was the reported risk factor for transmission for 22.7% of newly diagnosed HIV infections in the WHO European Region in 2021, with 48% of the infections among PWID being diagnosed late [2]. A history of injecting drug use is the most frequently reported risk factor for newly diagnosed acute and chronic viral hepatitis C (HCV) infections in the EU/EEA [3]. While the prevalence of infection with the hepatitis B virus (HBV) among PWID is lower than for HCV, it is still considerably higher than among the general population [4]. Finally, the prevalence of latent TB is higher among PWID who, due to a combination of social risk factors, malnutrition, tobacco use, problematic alcohol use and HIV-induced immunosuppression, are more likely to develop active TB with higher mortality outcomes [5, 6].

To meet the goal of the Global Viral Hepatitis Strategy [7] and the United Nations Sustainable Development Goal target 3.3 of ending the HIV and TB epidemics and combating viral hepatitis by the end of 2030 [8], PWID need to be prioritised for evidence-based interventions for each stage in the care cascade, from testing to curing hepatitis and TB or achieving viral suppression for HIV. However, the evidence base from peer-reviewed literature on interventions that could be applied in European settings has several limitations (e.g., poor quality study designs, small study population sizes, high selection biases) [9, 10]. In addition, there is an important geographical bias in the evidence, with only a limited number of studies published from EU/EEA and Eastern European countries. While often not published in peer-reviewed journals, the experiences of service providers for PWID can potentially be seen as a source of practice-based evidence where the impact of interventions is well documented. Particularly for hard-to-reach groups such as PWID, practice-based evidence can supplement gaps in the research with models of interventions implemented in real-life settings and with evidence of impact. In September 2020, the ECDC and EMCDDA launched a call for models of good practice (MoGPs) by reaching out to professional networks and key stakeholders in Europe with large geographical coverage. ‘Good practice’ was defined as an intervention or package of interventions that had shown evidence of effectiveness in particular settings and was likely to be replicable [11]. The benefit of collecting MoGPs that aim to improve health, thus sharing knowledge about interventions that work well in similar settings and populations, has been recognised by the European Commission as a promising tool for the transfer of expertise among Member States and the efficient use of resources [12].

### Study purpose

Faced with generally poor quality studies reporting on interventions in the care cascade for PWID identified in the systematic review [9], and a scarcity of research from countries in the EU/EEA and the Eastern European region, the aim of this study was to add practice-based evidence of interventions which optimise the care continuum (see Table 1) for infectious diseases in PWID. This was done by performing an in-depth, qualitative analysis of the interventions employed in 15 MoGPs in European settings included in the ECDC Models of good practice for community-based testing, linkage to care, and adherence to treatment for hepatitis B and C, HIV, and TB and for health promotion interventions to prevent infections among people who inject drugs [11]. The two research questions for the qualitative analysis were:

- What interventions were employed in the MoGPs for each stage in the care cascade (community-based testing, linkage to care, adherence to treatment), and what are their main implementation characteristics?
- What were the overarching enabling factors inherent in the implemented interventions?

## Methods

### Context and conceptual framework

The qualitative analysis utilises MoGPs selected following a call by the ECDC and EMCDDA to identify interventions implemented in real-life settings that can improve crucial stages in the care cascade, namely community-based testing, linkage to care and/or adherence to treatment, for HBV and HCV, HIV, and TB among PWID in Europe. This study is reported in accordance with the Standards for Reporting Qualitative Research (SRQR) [13]. Definitions of relevant terminology are provided in Table 1 below as well as in the supplementary material 1 (Table S1.1).

**Table 1:** Relevant terminology

• **Stages in the care cascade** are defined as the steps required to progress from the diagnosis of an infection or disease to treatment for viral suppression or disease cure [14, 15]. In the context of this study, the stages cover: (1) community-based testing, (2) linkage to care, and/or (3) adherence to treatment for HBV and HCV, HIV, and TB. The continuum of care aims at maintaining continuity of engagement of individuals from testing, linkage to care, and adherence to treatment and across multiple care and treatment facilities.	
• **Interventions** are efforts that aim to improve one or more stages in the care cascade (case management, contingency management, cooperation, decentralisation of services, directly observed therapy [DOT], integrated services, opioid agonist treatment [OAT], peers, telemedicine). The definitions of the interventions follow those used in the preceding systematic review by Schwarz et al. [9].	
• **Models of Good Practice (MoGP)** were defined as an intervention or a package of interventions that has shown evidence of effectiveness in particular settings and is likely to be replicable.	

### Collection and selection of models of good practice

*Call for expression of interest and submission*: Following an open call for expression of interest launched by the ECDC and the EMCDDA, national health authorities, including government and national infectious disease programmes, academics, public health/research institutes, and non-governmental organisations in EU/EEA or ENP countries were invited to submit examples of MoGPs through a standardised online reporting form (*EUSurvey* online tool. Available at https://ec.europa.eu/eusurvey) (see supplementary material 2, Table S2.1). The narratives of MoGPs were collected in September and October 2020. Submitters were invited to report on MoGPs that described interventions aiming at improving the following stages in the care cascade: (i) community-based testing for HBV, HCV, HIV and TB, (ii) linkage to care following diagnosis, and (iii) adherence to treatment for the infections. In addition, submissions were invited for health promotion interventions to prevent infectious diseases among PWID; details of the process and results can be found in the ECDC Report [11]. Details on the reporting form see supplementary material 2, Table S2.2.

#### Selection process

**Fig. 1:**
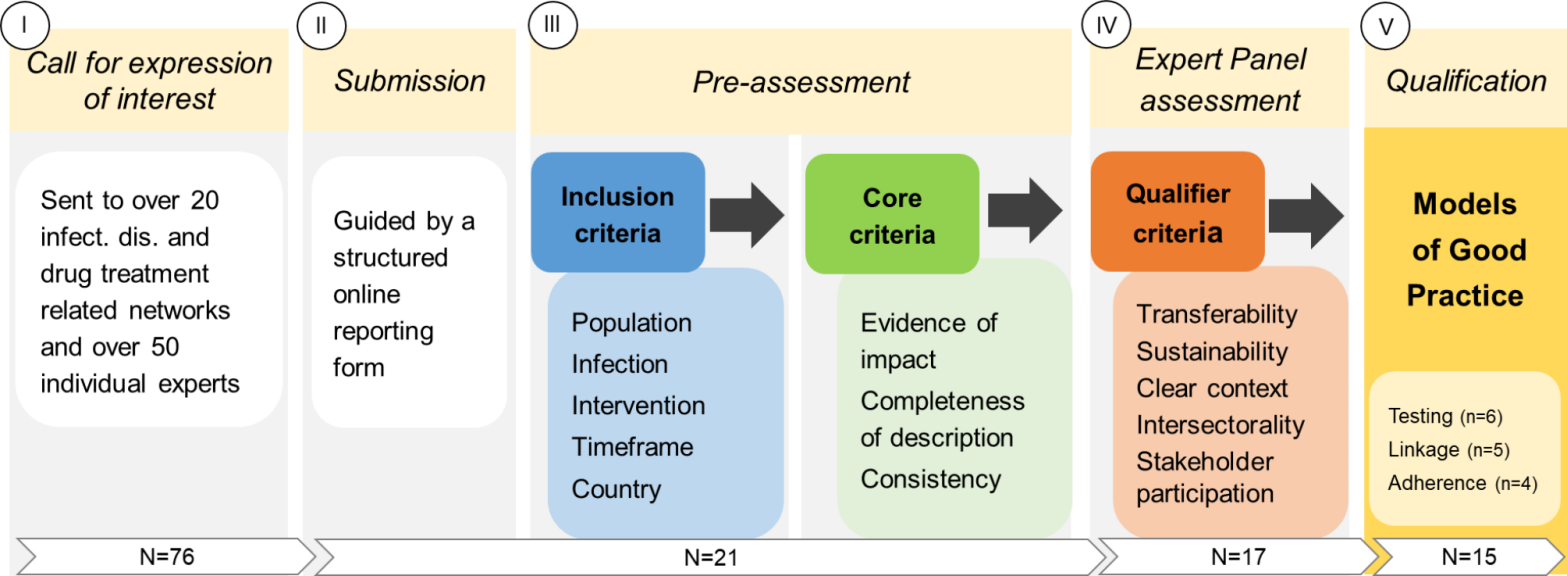
Overview of the standardised collection and selection process to identify MoGPs

The selection process was guided by a set of pre-defined criteria adapted from a European Commission’s Steering Group on Promotion and Prevention (SGPP) document [16]. It included a pre-assessment phase by the ECDC and GOEG and an assessment phase by an expert panel convened by the ECDC and EMCDDA to update the guidance for PWID [17] (Fig. 1). The expert panel’s terms of reference, areas of competencies covered and the outcomes of the consultation are extensively described in a technical report [17]. In the pre-assessment phase, the submissions (*n* = 21) were examined in relation to (i) *inclusion criteria* that aimed to evaluate the adequacy of the intervention based on its relevance/ characteristics, and (ii) *core criteria* that considered whether the intervention was successful and had a documented impact (see column III in Fig. 1). Following (i) and (ii), a total of 17 submissions were considered eligible for the next step in the selection process, namely an assessment by members of the expert panel that looked at (iii) *qualifier criteria* such as transferability to other settings, sustainability, a clearly described context, as well as aspects of intersectorality and stakeholder participation (see column IV in Fig. 1). Fifteen submissions were selected as MoGPs, six addressing community-based testing for HBV, HCV, HIV and TB, five addressing linkage to care following diagnosis, and four referring to adherence to treatment for the infections concerned. The assessment form can be found in the supplementary material (Table S2.2).

### Data analysis

#### Preparatory steps

Prior to data extraction, we agreed on interventions already used in the systematic review (case management, contingency management, cooperation, decentralisation of services, directly observed therapy (DOT), integrated services, opioid agonist treatment (OAT), peers, telemedicine) to look for and we used their definition as formulated in the systematic review [9]. Each of the 15 MoGPs selected for analysis was given a label consisting of one letter, A, B or C, referring to a stage in the care cascade (A community-based testing, B linkage to care, C adherence to treatment) and one sequential number (e.g., A.1, B.3, C.4; see Table 1). Specific terminology is described in the glossary (see supplementary material 1, Table S1.1). The indicators documenting effectiveness of MoGPs were listed systematically as reported by submitters by stage in the care cascade and for each infectious disease (see supplementary material 2, Table S2.1). The diversity of indicators used in each MoGP prevented a systematic pooling (see the full list of effectiveness indicators utilised in the MoGPs in ECDC technical report [11].

#### Descriptive analysis of the MoGPs

To provide a general overview on the selected MoGP, descriptive data on each MoGP (e.g., country, geographical coverage, infections targeted, and settings were extracted by two authors (IH, TS) and recorded in three Excel spreadsheets, one for each stage in the care cascade. Characteristics of each MoGP were summarised based on frequency.

#### Qualitative content analysis of interventions

**Fig. 2:**
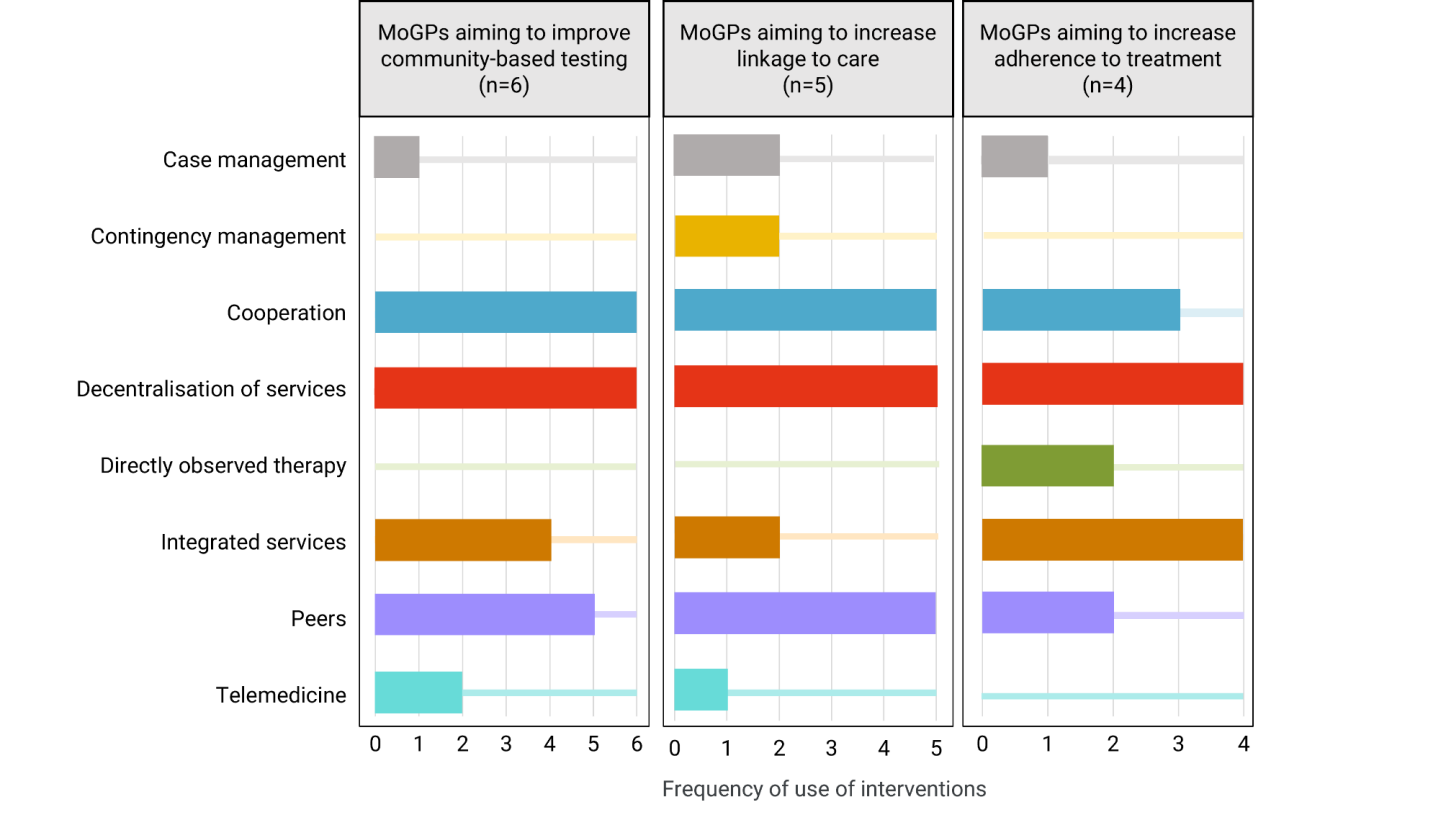
Type of interventions identified across the MoGPs (*n* = 15) and their frequency of use in each stage of the care cascade. *Interventions as defined in Schwarz et al.* [9]

To answer the first research question, information was extracted by one author (IH) for each MoGP resulting in (i) a quantified overview of types of interventions and (ii) three Excel spreadsheets where implementation characteristics across all MoGPs were organised by each stage in the care cascade (see Fig. 2). The extracted information was used for the qualitative content analysis that aimed to synthesize implementation characteristics, using pre-defined types of interventions (deductive approach, P Mayring [18]).

#### Qualitative content analysis of enabling factors

**Fig. 3:**
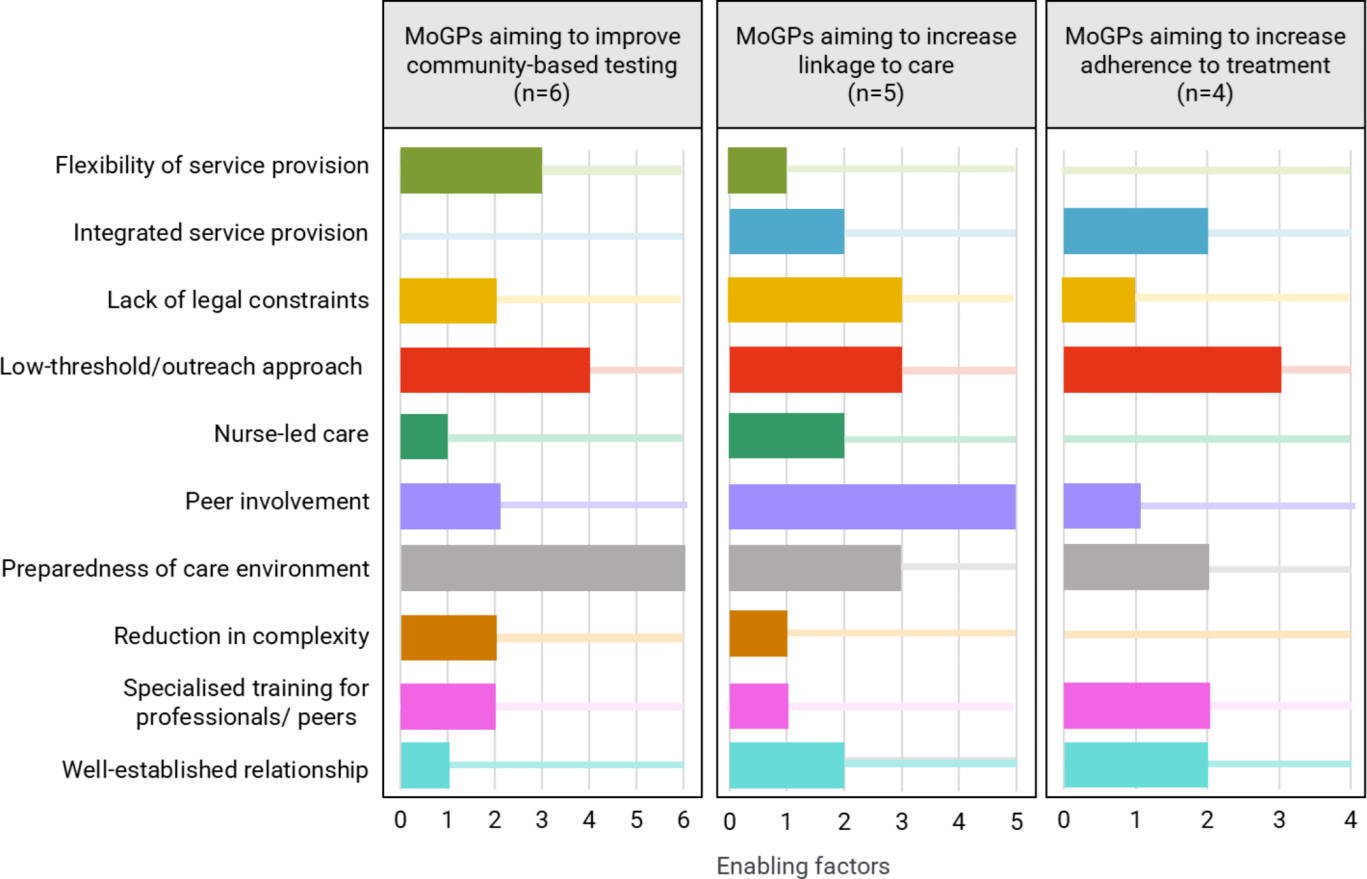
Enabling factors included in MoGPs (*n* = 15) for each stage of the care cascade including frequency. *Note: preparedness of care environment refers to awareness of service providers and existence of effective pathways and communication channels*

To answer the second research question, one author (IH) extracted information on enablers of interventions as reported by MoGPs submitters when answering question 4.2 in the reporting form (‘What are – in your opinion – the main factors contributing to the success of the intervention?’) and recorded it in an Excel spreadsheet. Next, extracted information on the enabling factors was grouped for each stage in the care cascade, following an inductive category development [18], resulting in: (i) quantified categories of enabling factors (see Fig. 3), and (ii) a more detailed description of the characteristics of these factors. As a quality check, TS and OM reviewed the extracted text material and the quantification.

## Results

An overview of the 15 MoGPs with detailed information on the country, project, type of interventions used, infections targeted, and the implementation setting is presented in Table 2. The MoGPs were implemented under ten different national projects in six countries, Belarus (1), Norway (1), Portugal (2), the Republic of Moldova (1), Spain (1), the UK (3), and in the multi-country HepCare project carried out in Ireland, Romania, Spain, and the UK. As Fig. 2 indicates, a package of interventions was used in all stages of the care cascade to effectively improve testing for infections, linkage to care and adherence to treatment among PWID.

**Table 2:** Synopsis of Models of good practice (MoGPs) (*n* = 15) by country (*n* = 8) and project (*n* = 10) with details on infection targeted, implementation settings and type of interventions used for each stage in the care cascade

Country	Title of project (national or multi-country)	Affiliation of submitting author/ organisation	Infection(s) targeted	MoGPs ID*	Implementation setting for each stage			Type of interventions used for each stage		
					Community-based testing (A)	Linkage to care (B)	Adherence to treatment (C)	Community-based testing (A)	Linkage to care (B)	Adherence to treatment (C)
Republic of Moldova	Accelerating the TB/HIV response for key populations in EECA cities	Union for HIV Prevention and Harm Reduction	HIV/AIDS, TB	A.1	Harm reduction service, OAT setting, outreach**	*N/A*	*N/A*	Decentralisation, Peers, integrated services, cooperation	*N/A*	*N/A*
Portugal	REACH-U – Point-of-care hepatitis C antibody, RNA testing and linkage to care to enhance uptake of treatment in outreach settings	CRESCER - Associação de Intervenção Comunitária	HCV	A.2 B.3 C.3	Harm reduction service, outreach	Harm reduction service, outreach	Harm reduction service, outreach	Decentralisation, Peers, Cooperation, telemedicine	Decentralisation, Peers, Cooperation, Telemedicine, contingency management	Decentralisation, Peers, integrated services, cooperation, DOT
Belarus	Improvement in HIV testing rates and involvement of PWID in dispensary observation at prevention points in Minsk	Belarusian Public Association “Positive Movement”	HCV***, HIV/AIDS	A.3 B.1	Harm reduction service	Harm reduction service, OAT setting, outreach	*N/A*	Decentralisation, integrated services, cooperation, case management	Decentralisation, peers, cooperation, case management, contingency management	*N/A*
Norway	Hepatitis C Bus (HCV bus)	Norwegian Directorate of Health	HCV, HIV/AIDS	A.4	Harm reduction service, OAT setting, Prison, outreach	*N/A*	*N/A*	Decentralisation, Peers, cooperation	*N/A*	*N/A*
Portugal	Mobile Outreach Programme	Associação Ares do Pinhal	HBV, HCV, HIV/AIDS, TB	A.5 B.2 C.4	Harm reduction service, OAT setting, outreach	Harm reduction service, outpatient treatment centre	Harm reduction service; OAT setting, outreach	Decentralisation, Peers, integrated services, cooperation	Decentralisation, Peers, integrated services, cooperation	Decentralisation, Peers, integrated services, cooperation, DOT
UK	Find & Treat: Peer-led Blood Borne Virus Community Outreach Project	Find & Treat, UCLH NHS Trust/ Institute of Global Health	HBV, HCV, HIV/AIDS, TB	A.6	Outreach	*N/A*	*N/A*	Decentralisation, Peers, integrated services, cooperation, telemedicine	*N/A*	*N/A*
Spain	Comprehensive care of patients with substance use disorders	Addiction Research Group, Institut Hospital del Mar d’Investigacions Médique, Barcelona	HIV/AIDS	C.1	*N/A*	*N/A*	Outpatient treatment centre	*N/A*	*N/A*	Decentralisation, integrated services, cooperation, case management,
UK	Hepatitis C Elimination Programme	NHS England and NHS Improvement	HCV	B.4	*N/A*	OAT setting, Prison, Outreach, pharmacies	*N/A*	*N/A*	Decentralisation, Peers, Cooperation,	*N/A*
UK	Engaging the disengaged: ITTREAT, VALID	Brighton and Sussex Medical School and Brighton and Sussex University Hospital, Brighton	HCV	B.5	*N/A*	Outreach	*N/A*	*N/A*	Decentralisation, Peers, integrated services, cooperation, case management	*N/A*
Ireland, Spain, Romania, UK	HepLink arm of the HepCare Europe project	University College Dublin	HCV	C.2	*N/A*	*N/A*	Outpatient treatment centre, OAT setting	*N/A*	*N/A*	Decentralisation, integrated services

* Note: The classification is based on the ECDC framework of the call, and the allocation is based on the submitter’s choice. An identification code (ID) was given to each MoGP with the letter corresponding to the stage in the care cascade: A = community-based testing, B = linkage to care and C = adherence to treatment; the number represents the current number assigned to the MoGP submission. ** Note: outreach refers to outreach programmes. *** Note: in this MoGP, HCV is only addressed in community-based testing

### Optimising community-based testing for infections among PWID

The six MoGPs that aimed at improving community-based testing for infections among PWID were reported from the Republic of Moldova (A.1), Portugal (A.2, A.5), Belarus (A.3), Norway (A.4), and the UK (A.6). Three (A.1, A.2, A.3) had local coverage, and three (A.4, A.5, A.6) regional coverage. One MoGP aimed at optimising community-based testing for HCV (A.2), two for HCV and HIV/AIDS (A.3, A.4), and one for HIV/AIDS and TB (A.1); two looked at integrated testing for HBV, HCV, HIV/AIDS, and TB (A.5, A.6).

#### Settings

Most interventions (A.1, A.2, A.4, A.5) were implemented in more than one setting (see Table 1), reaching out to PWID for testing through harm reduction services (A.1, A.2, A.3, A.4, A.5), by means of outreach work (A.1, A.2, A.4, A.5, A.6), an OAT setting (A.1, A.4, A.5), and prison (A.4).

#### Type of interventions and their characteristics

The following pre-defined interventions were identified in the MoGPs: decentralisation, peers, integrated services, cooperation, case management, and telemedicine (see Fig. 2).

To optimise community-based testing all MoGPs used decentralised specialised services (A.1-A.6; see Table 1). Decentralised testing included outreach and low-threshold screening for infections free-of-charge (A.1-A.6); in some cases, it also covered confirmatory testing and specific clinical examinations, e.g., fibrosis stage, or mobile x-ray technology to identify active TB cases (A.2-A.6). Testing at point-of-care (POC) (A.2, A.6) aimed not only to facilitate access to testing but also to communicate results (via text message or team members) in a timely manner. POC testing was often connected to needle and syringe programmes (NSP) and other low-threshold drug services (A.2, A.3, A.5).

Peer interventions played an essential role in community-based testing in five MoGPs (A.1, A.2, A.4, A.5, A.6; see Table 1). Peers were involved in outreach to provide direct support to PWID and improve participation in screening (A.1, A.2, A.4, A.6). They also acted as facilitators for interacting with and building trust in services provided (A.1, A.5) and were involved in peer-to-peer training raising awareness of blood-borne viruses (A.6), on POC testing and on FibroScan® (as defined in NH Afdhal [19]) (A.2, A.6). Two MoGPs reported using peer-to-peer recruitment methods for mobilisation (A.4, A.1). Peers were seen as team members in one MoGP, helping to adapt the conceptualisation of testing activities to better meet the needs of PWID (A.5). One MoGP mentioned specific training as well as clinical supervision for involved peers (A.6).

An integrated one-stop-shop approach was indicated by four MoGPs aiming to optimise community-based testing (A.1, A.3, A.5, A.6). This included multiprofessional teams providing various services to PWID, from infectious disease screening to medical supervision (A.1, A.3, A.6), from public health to social support (A.3, A.6), sometimes combined with OAT settings (A.1, A.5) or outreach work (A.5). Cooperation between low-threshold services and local hospitals, primary healthcare centres and social services was considered an effective intervention in all MoGPs aiming to increase community-based testing (A.1-A.6). Case management was only reported in one MoGP with the aim of ensuring continuity of care after the initial contact (A.3). Telemedicine approaches were used to facilitate appointments with specialists after testing (A.2, A.6).

### Optimising linkage to care of PWID testing positive for infections

The five MoGPs that aimed at increasing linkage to care for infections among PWID were from Belarus (B.1), Portugal (B.2, B.3), and the UK (B.4, B.5). Two had local coverage (B.1, B.3), two were regional (B.2, B.5), and one national (B.4). Three MoGPs (B.3, B.4, B.5) addressed HCV, one targeted HIV/AIDS (B.1), and one addressed linkage to care for several infections/comorbidities, HBV, HCV, HIV/AIDS, and TB (B.2).

#### Settings

Most interventions targeted PWID in more than one setting (B.1, B.2, B.3, B.4) including harm reduction services (B.1, B.2, B.3), outreach programmes (B.1, B.3, B.4, B.5), outpatient drug addiction treatment centres (B.2), OAT setting (B.1, B.4), prison (B.4) and pharmacies (B.4).

#### Type of interventions and their characteristics

Interventions involving the decentralisation of specialised services (see definition in supplementary material 1) were used by all MoGPs that aimed to improve linkage to care of infections among PWID by facilitating initial appointments with a specialist in outreach settings (B.1-B.5; see Fig. 2). To enhance treatment initiation and uptake, all five MoGPs provided as many services as possible within the community (B.1-B.5). For instance, clinical assessments for PWID testing positive for infections or initial appointments with a specialist following a positive test were offered on site at places that best meet the person’s needs (B.2, B.3, B.4). These can be either low-threshold drug services (B.2, B.3, B.4) or, in the context of outreach and depending on the possibility of cooperation among providers, in shelters, prisons, community pharmacies or OAT settings (B.3, B.4). If transfer to a hospital was necessary, efforts were made to bridge the gap between PWID’s life realities such as ongoing substance use or unstable housing and hospitals’ highly rigid routines with the help of nurse- or peer-led outreach activities (B.1, B.2, B.3) and flexible drop-in clinic appointments (B.5). Telemedicine was used to organise online appointments with clinical specialists (B.3).

Peer interventions were included in all MoGPs aiming to optimise linkage to care, indicating their important role in scaling up treatment initiation (B.1-B.5). In the outreach context, responsibilities included helping to trace the target population (B.1, B.4), facilitating contacts, in particular with hard-to-reach groups within the PWID population (B.3, B.4), fostering trust in the healthcare system (B.3, B.4) and providing support for treatment initiation (B.3, B.4, B.5). Peer navigators eased barriers to specialist services and supported transfers to hospitals when needed (B.2, B.4, B.5). One MoGP involved peers in programme development to better meet the needs of the target population (B.2).

In all MoGPs there was a close cooperation between service providers (B.1-B.5), particularly low-threshold drug services and local hospitals or other healthcare facilities (B.1, B.2, B.3, B.5). To increase reachability, some MoGPs also worked with local shelters (B.4, B.5), primary care (B.5), local pharmacies (B.2, B.4, B.5) and laboratories (B.5) or national HCV elimination programmes (B.4, B.5). Specialised training was offered aiming to increase awareness of drugs and consumption patterns and/or training on infectious diseases among professionals and/or their partner institutions (B.3, B.5) as well as training for peers (B.4).

Integrated service approaches to increase linkage to care were mentioned in two MoGPs (B.2, B.5), one related to multiprofessional teams comprising medical practitioners, nurses, psychologists and social workers (B.2) and the second to a low-threshold, one-stop-shop concept aiming to provide everything necessary for treatment initiation on one site (B.5). Case management interventions were implemented by two MoGPs (B.1, B.5) through outreach contacts (B.1) or the permanent availability of specialised staff via mobile phone (B.5), with another two using contingency management (provision of vouchers or cash) to stimulate attendance at the first appointment (B.1, B.3).

### Optimising adherence to treatment for Infections among PWID

The four MoGPs that aimed at increasing adherence to treatment for infections among PWID were from Spain (C.1), Portugal (C.3, C.4), and the multi-country project (C.2) implemented in Ireland, Romania, Spain, and the UK. Three had local coverage (C.1, C.2, C.3) and one was at regional level (C.4). Two MoGPs addressed adherence to HCV treatment (C.2, C.3), one targeted HIV/AIDS (C.1), and another adherence to integrated treatment for PWID with several infections and/or comorbidities (C.4).

#### Settings

Interventions were implemented in one or more of the following: outpatient drug treatment centres (C.1, C.2), OAT sites (C.2, C.4), outreach programmes (C.3, C.4), and low-threshold harm reduction services (C.3, C.4).

#### Type of interventions and their characteristics

Providing integrated services was the aim of all four MoGPs to help optimise adherence to treatment (C.1-C.4; see Fig. 2). Apart from infections (e.g., HCV, HIV), PWID were also treated for substance use disorders, i.e., OAT (C.1, C.2), co-infections and/or psychiatric comorbidity (C.1) or provided with psychosocial support and general healthcare services (C.1, C.2, C.4). Three MoGPs involved multidisciplinary teams including internal medicine specialists, psychiatrists, general practitioners, social workers, nursing staff and psychologists (C.1, C.3, C.4). Peers were also included in the multidisciplinary teams (C.3, C.4).

The decentralisation of treatment was mentioned by all MoGPs to help increase adherence to treatment for infections among PWID (C.1-C.4). Two MoGPs set up a community-based medical procedure based on an existent low-threshold service, including on-site dispensing of medication by mobile outreach units (C.3, C.4). In the other two MoGPs, infectious disease treatment initiatives cooperated with drug treatment services such as OAT, also embedding the treatment of infections in a low-threshold setting to overcome barriers to treatment in PWID (C.1, C.2). Outreach of HCV-trained nurses into primary care and community settings was another approach described (C.2).

Peer interventions to promote adherence to treatment were mainly used to foster trust in healthcare and to facilitate interactions, particularly between current PWID and service providers (C.3, C.4). In one MoGP (C.4), peers were also involved in the design of treatment service set-ups aiming to best meet the needs of PWID. Cooperation between providers was used to increase adherence to treatment by three MoGPs (C.1, C.3, C.4), including drug treatment services (C.1, C.4) and/or low-threshold drug services (C.3, C.4), local healthcare providers (C.1, C.4) or hospitals (C.3, C.4) and potentially local pharmacies to provide medication (C.1). One MoGP cooperated with shelters, prisons and social centres to deliver treatment regimens (C.4). A person-centred approach, also referred to as case management, was used in one MoGP (C.1) where the nursing staff reviewed the potential side effects of therapy with patients, identified adherence difficulties to help secure retention in treatment, provided education on adherence and even reached out for the clients if they did not attend their appointments. Peers were involved in two projects conducted in low-threshold services (C.3, C.4) which also used DOT to dispense medication (C.3: direct-acting antivirals [DAA] for HCV; C.4: not specified).

#### Enabling factors to improve the impact of interventions

As an overarching success factors for interventions addressing the PWID population, enablers address structural or social factors while others refer directly to specific types of interventions used. Figure 3 provides an overview of the enablers synthesised for each stage of care cascade.

The preparedness of care environments, in terms of awareness of service providers and existence of effective pathways and communication channels, facilitated cooperation among service providers while functional connections between the principal actors (e.g., low-threshold services, local authorities, medical and social institutions) were considered an enabling factor in all stages of the care cascade (A.1-A.6; B.2, B.4, B.5, C.3, C.4). These pre-existent working structures mentioned by eleven out of the 15 MoGPs, support a coordinated effort with relevant stakeholders (A.*), create the foundations for successful linkage to care (B.*) and build the backbone for provision of specialist treatment in outreach settings (C.*). Four MoGPs (A.2, A.5, B.3, C.4) emphasised both the lack of legal constraints, in terms of general political consent to provide the care cascade to PWID, and the wide availability of harm reduction services as basic conditions for a successful testing-to-treatment pathway. In view of the multiple health and social needs of PWID, providing integrated services and involving multidisciplinary teams were highlighted in four MoGPs as enabling factors for linkage to care (B.2, B.5) and adherence to treatment (C.1, C.4).

A low-threshold approach to reaching out for PWID was emphasised as important to overcome barriers along the care cascade, in particular for interventions aiming to improve (community-based) testing and linkage to care. For the former (A.3-A.6), availability of POC rapid testing was perceived as added value while for the latter (B.1, B.2, B.5), the low-threshold approach was seen as a facilitator for transfer to specialist appointments. In terms of adherence to treatment, community-based and outreach treatment approaches (C.1, C.4, C.3) were described as enablers, bringing clinical treatment closer to the lived realities of PWIDs. It was pointed out that this relates closely to the flexibility of service providers increasing their reachability for PWID (A.2, A.3, A.6) as well as to a reduction in complexity in terms of the number of service sites clients need to visit along the care cascade (A.3, A.6). For linkage to care, healthcare services either established flexible drop-in appointments for PWID (B.3) or one-stop-shop models offered multiple specialist services at the same place (B.5).

Availability of specialised training to staff or peers was considered an enabler in MoGPs for all three stages of the care cascade. While the training content was not specified for the interventions targeting community-based testing (A.2, A.6), in linkage to care interventions, training on substance misuse was offered to community hepatitis nurses (B.5) and for adherence to treatment, it covered addiction medicine and/or HCV infection treatments (C.1, C.3).

Access to free of charge treatment as an important structural prerequisite was mentioned in one MoGP (C.1).

In the MoGPs, use of peers was mentioned in a double function: as intervention to optimise the progression of PWID along the care cascade (A.1, A.2, A.4, A.5, A.6, B.1-B.5, C.3, C.4), and as facilitator in all stages of the care cascade (A.1, A.6, C.3) but in particular when increasing linkage to care (B.1-B.5). As such, the inclusion of “experts by experience” facilitates access to specialist appointments (B.1-B.5), helping to overcome existing barriers in the care cascade and building trust in the healthcare system. Nurses (e.g., hepatitis nurses) deployed in outreach settings were identified as enablers in three MoGPs (A.3, B.1, B.5), helping to lower the threshold for medical services in the care cascade.

The well-established relationship of trust between service providers and PWID resulting from long-term contact was described as the foundation for interventions optimising the care cascade. These relationships were seen to be the result of preparatory work by professionals aiming to establish trust between patients and professionals (A.5, B.1, B.2, C.1, C.4). The highly intrinsic motivation of professionals (C.1) was also mentioned as an enabling factor.

## Discussion

We undertook an in-depth, qualitative examination of 15 MoGPs implemented in EU/EEA and ENP settings with the aim of identifying interventions that can optimise community-based testing, linkage to care and adherence to treatment for hepatitis B and C, HIV, and TB among PWID. The ten projects (national and/or multi-country) from where the 15 MoGPs emerged, addressed all stages in the care cascade, with HCV targeted by most (8/10), followed by HIV/AIDS (6/10), TB (3/10), and HBV (2/10).

The qualitative analysis of interventions employed in the MoGPs revealed benefits in the care cascade when several interventions are combined to achieve synergies and implemented in multiple settings to enhance their reachability for PWID. The decentralisation of testing and treatment services for PWID and collaboration between care providers are the two interventions reported across all stages in the care cascades and in all but one MoGP settings (the exception being cooperation). Likewise, a recent systematic review and meta-analysis [20] with worldwide coverage adds evidence that decentralising HCV care to non-specialised services, such as harm-reduction sites or primary care, improved access to testing and linkage to care. Dispensing HCV treatment through non-specialised services was associated with similarly high rates of cure as those achieved in specialised clinics. The results of the multi-country HepCare project in Europe [21] showed that collaboration between community‐based organisations and specialised care services to recruit PWID for HCV screening, to ensure their linkage to care and support their adherence to treatment, can probably be reproduced on a larger scale.

The one-stop shop approach, an integrated care model combining multispecialised teams to gather all necessary competences on one site, can reduce the complexity of care pathways [22]. These interdisciplinary, integrated care structures consistently show positive effects on the health and wellbeing of PWID, including mental health outcomes [15, 23, 24], by reducing the risk of loss to follow-up when patients have to navigate through complicated standard healthcare pathways [25]. Our analysis shows that integrated approaches that included testing, medical supervision, peer counselling, social support and/or OAT provided on one site were of particular importance for increasing adherence to treatment (4/4 MoGPs) and also for optimising community-based testing (4/6 MoGPs).

Peer involvement is another integrative part in the majority of MoGPs, especially those targeting community-based testing and linkage to care. The inclusion of “experts by experience” has also been identified as an enabling factor in linkage to care interventions. This is consistent with the findings from a systematic integrative review by Bouzanis et al. [24] reporting that peer-based services can improve delivery of care for PWID and address issues such as lack of trust and unfamiliarity with the healthcare system and healthcare professionals.

Telemedicine was employed in MoGPs addressing community-based testing and linkage to care, especially to support access to care and facilitate communication for PWIDs or patients living in remote areas. This is in line with Jiménez Galán et al. [26], who concluded that telemedicine can facilitate referrals to specialists. Further research should explore how telemedicine approaches may improve the retention in care of certain PWID subgroups and how telehealth options can be incorporated effectively in integrated care structures. Contingency management to improve the cascade of care was only implemented in two MoGPs (Belarus and Portugal) to increase linkage to care; both used incentives in combination with other interventions. This matches findings from the systematic review by Schwarz et al. [9], which stressed that contingency management can facilitate linkage to care of PWID but is less likely to ensure adherence to treatment.

None of the MoGPs indicated enrolment in OAT as a prerequisite for receiving treatment for an infectious disease. The most recent EASL guidelines for treating HCV highlight that PWID should have access to HCV treatment (DAA) regardless whether they receive OAT [27]. OAT programme sites were, however, mentioned as settings to reach out to PWID and successfully manage individualised treatment regimens [28].

Our analysis identified several enabling factors reported to influence the success of interventions implemented in the MoGPs. Effective interventions can only be implemented when systemic barriers to infectious disease treatment for PWID are removed. Adequate funding should be assured, testing and treatment should be free of charge and recent drug use should not be an exclusion criterion for eligibility for treatment [17, 27]. The MoGPs documented that the interventions need to be tailored to the structure of national (health) care systems as well as national legal frameworks. Endorsement from the government (local or national authorities) and proof of political will is key for implementing interventions.

A further essential prerequisite for successful interventions concerns the preparedness of care structures, including the presence of staff sensitised to assisting PWID and existing collaborations between various types of service providers and settings in which treatment is delivered (e.g., pharmacies already involved in OAT). The importance of considering social and structural factors that may impede infectious disease care for PWID is also emphasised by other studies [24, 29].

Looking at the social dimension of enablers, qualified professionals, cooperation among partners and peer-involvement approaches are prerequisites to address PWID in their lived realities and meet their particular needs. As indicated by Amoako et al. [30] and Marshall et al. [31], appropriate education, training, and knowledge exchange among providers will reduce challenges that staff can encounter when treating PWID, including that of treating patients with complex comorbidities, mental health issues, low social support, housing insecurity, and/or drug use that could potentially interfere with the treatment plan. Where decentralisation of care is not successful or feasible for various reasons, low-threshold organisations and services can help PWID overcome systemic barriers. Involving peers, outreach nurses and other social (health) care workers can act as a lever to accompany clients throughout the entire referral process [17].

The rigour of our results and the certainty that the interventions reported from practice are effective were enhanced by including in the analysis only MoGP selected based on a standardised assessment process with a set of pre-defined quality criteria. Transferability and sustainability of the MoGPs in other European settings were assessed by an independent expert panel with a wide European geographical coverage. This process aligns with the approach recommended by the European Commission in its DG SANTE guide to identify and transfer best practices between Member States [16]. However, some **limitations** apply to our analysis. First, the MoGPs included are based on voluntary responses to a call for submissions and not on a comprehensive mapping of practices based on the full participation of all invited networks and stakeholders. Second, some MoGPs addressed more than one stage in the care cascade through packages of interventions. We only included information in the analysis that directly related to the field of intervention for which it had been submitted; interventions in some MoGPs may overlap with interventions of other implementation settings, showing the continuous nature of the cascade of care. Third, information on training for peers involved in cooperation networks was insufficient as details were not requested as part of the reporting form. Fourth, the information provided did not allow an in-depth analysis of differences in care and treatment approaches for each distinctive infection.

Several important research gaps and **implications for future research** were identified. Enhancing the care cascade by including policy-level, care structure-based and socially determined prerequisites for successful care and treatment requires urgent attention in research agendas. Large-scale comparative studies and qualitative research into implementation practices are required to gain further evidence of valuable practice-based experiences supporting improved inclusion of PWID in treatment care cascades and tailor future strategies to prevent and treat infectious diseases among highly vulnerable groups in EU/EEA countries and elsewhere.

## Conclusion

MoGPs selected from European settings demonstrate that combination interventions, and the involvement of multiple sectors of service providers are successful at improving testing, linkage to care, and adherence to treatment for infectious diseases among PWID. The interventions were designed to overcome the particular patient-, provider- and system-level barriers impacting the engagement of PWID with infectious disease care. The results highlight that care structures need to be (i) simplified (by reducing complex testing and treatment pathways), (ii) based on cooperation (in a particular facility or in collaboration with other services already utilised by PWID), and (iii) based on multiprofessionality (comprising healthcare professionals, social healthcare workers, experienced outreach workers, trained peers, and more). MoGPs allow knowledge exchange about new approaches to improve health outcomes among PWID, and provide additional evidence for policy makers, public health researchers, and (inter-)national programme coordinators involved in the prevention and control of infectious diseases among highly vulnerable groups.

### Electronic supplementary material

The link to the electronic supplementary material is provided below. 10.1186/s12913-023-10412-y

Supplementary Material 1: Table S1.1 Glossary of interventions and intervention component definitions.

Supplementary Material 2: Table S2.1 Models of good practice: Online reporting form for interventions to increase stages of care cascade among PWID (A-C). Table S2.2 Models of good practice: Assessment form for interventions to increase stages of care cascade (A-C).

## Data Availability

The datasets generated and/or analysed during the current study are partly published in ECDC [11] and available from the corresponding author on reasonable request.

## Abbreviations

AIDS: Acquired immunodeficiency syndrome
DAA: Direct-acting antivirals
DOT: Directly observed therapy
EU/EEA: European Union/European Economic Area
ENP: European neighbourhood policy
GOEG: Gesundheit Österreich GmbH (Austrian National Public Health Institute)
HBV: Hepatitis B virus
HCV: Hepatitis C virus
HIV: Human immunodeficiency virus
MoGP(s): Model(s) of good practice
NSP: Needle and syringe programmes
OAT: Opioid agonist treatment
POC: Point-of-care
PWID: People who inject drugs
RCT: Randomised controlled trial
SVR: Sustained virological response
TB: Tuberculosis
