# Supplementary material for: Models of good practice to enhance infectious disease care cascades among people who inject drugs: a qualitative study of interventions implemented in European settings
Source: BMC Health Serv Res. 2023 Dec 4;23:1352. doi: 10.1186/s12913-023-10412-y (PMC10696743; doi:10.1186/s12913-023-10412-y)
Supplement: Supplementary file 1 — Supplementary Material 1: Table S1.1 Glossary of interventions and intervention component definitions. [file 12913_2023_10412_MOESM1_ESM.docx]

**Supplementary material 1**

**Table S1.1 Glossary of interventions and intervention component definitions**

| **Component** | **Definition** | |
| --- | --- | --- |
| **Adherence to treatment** | The extent to which a patient actively follows through with prescribed medical treatment and recommendations (ECDC/EMCDDA, 2023). |  |
| **Case management** | Case management is a person-centred approach to improve the coordination and continuity of service delivery, especially for vulnerable clients with multiple needs, like people who inject drugs (Vanderplasschen, Rapp, De Maeyer, & Van Den Noortgate, 2019). In regard to infectious diseases, it supports clients by increasing awareness of the need for and access to all steps within the continuum of care. The role of a case manager is often undertaken by a trained nurse or social worker (Feder, Klinedinst, & Yee, 2018). |  |
| **Community-based testing** | Programmes and services that offer voluntary, free and anonymous infection testing outside formal health facilities designed to target specific communities (ECDC/EMCDDA, 2023). |  |
| **Contingency management** | A behavioural management technique that involves using incentives such as cash, vouchers, prizes or other kinds of privileges to reinforce behaviours or disincentives to discourage them (ECDC/EMCDDA, 2023). |  |
| **Continuum of care** | Continuum of care describes steps in the patient pathway (ECDC/EMCDDA, 2023). It aims at maintaining continuity of engagement of individuals from testing, linkage to care and adherence to treatment and across multiple care and treatment facilities. |  |
| **Cooperation** | Cooperation refers to a systematic collaboration between health care service facilities/organisations, including drug services, in the delivery of care and treatment (Boon, Mior, Barnsley, Ashbury, & Haig, 2009). |  |
| **Decentralisation of services** | Decentralisation of services refers to the delivery of infectious disease treatment at peripheral health facilities, community-based venues and low-threshold locations, meeting the specific needs of hard-to-reach populations, whereas centralised services are delivered at hospital sites or specialised clinical settings (WHO, 2018). |  |
| **Directly observed therapy** | A method of treatment administration in which a healthcare professional watches as a person takes each dose of a medication to ensure all medications are received and taken as prescribed (ECDC/EMCDDA, 2023). |  |
| **Integrated services** | Integrated care in the context of infectious diseases among people who use drugs is a person-centred combination of services required to meet the specific needs of the individual. Integrated care aims to ensure continuity of care. It includes treatment, care and psychosocial support to be inclusive and holistic. The service entails being needs-led and integrating organisational or administrative practices to facilitate the transition between specialised clinical treatment and community-based drug service providers. Prerequisites for integrated care are collaborative work between service providers as well as the development of multidisciplinary teams and/or care networks (Goodwin, 2016; Scottish Executive, 2002). Integrated services feature a network of interlinked support (EMCDDA, 2020). |  |
| **Interventions** | Interventions are efforts that aim to improve one or more stages in the care cascade that were reported on in the MoGPs identified (case management, contingency management, cooperation, decentralisation of services, directly observed therapy [DOT], integrated services, opioid agonist treatment [OAT], peers, telemedicine). |  |
| **Low-threshold services** | Social and health services for people who use drugs that typically offer a place to be, health education and counselling, needle and syringe programmes, referral to drug treatment, and sometimes overnight shelter. The aim is to make help more easily accessible and to remain in contact with individuals who have difficulty accessing and/or remaining in care (ECDC/EMCDDA, 2023). |  |
| **Linkage to care** | The process that links a person newly diagnosed with an infectious disease to care, including medical treatment (ECDC/EMCDDA, 2023). |  |
| **Models of good practice (MoGP)** | An intervention or a package of interventions that has shown evidence of effectiveness in particular settings and is likely to be replicable (ECDC, 2023) |  |
| **Nurse-led care** | In this model, a nurse is responsible for the overall coordination, management and continuity of care, including testing and/or treatment (Cullum, Spilsbury, & Richardson, 2005). |  |
| **Outreach programme** | Outreach is a systematic approach to delivering harm reduction services to people who inject drugs and to their sex partners/spouses in their own environments (UNODC, 2012). |  |
| **Opioid agonist treatment (OAT) programme** | Use of opioid agonist medications such as methadone and buprenorphine to prevent withdrawal symptoms and reduce drug cravings among opioid-dependent individuals. OAT is also referred to as opioid agonist maintenance treatment (OAMT) by the World Health Organization (ECDC/EMCDDA, 2023). In this study, OAT is seen as a setting and not as an intervention. |  |
| **Peers** | Persons within a community with equal standing with each other, belonging to the same group and sharing a common experience. Peer support, which can occur informally or formally, refers to support provided and received by people who are peers (ECDC/EMCDDA, 2023). |  |
| **People who inject drugs (PWID)** | Individuals who inject any psychoactive substance not according to medical prescription. In the guidance, the recommendations for using this term may also include individuals on OAT (ECDC/EMCDDA, 2023). |  |
| **Point-of-care tests (POC)** | Tests that are performed near the patient and on any part of the patient's body or its derivatives, during or very close to the time of consultation, allowing results at the time of clinical decision making to support clinical decision making (ECDC/EMCDDA, 2023). |  |
| **Screening** | The presumptive identification of unrecognised disease or defect by the application of tests, clinical examinations or other procedures that can be applied rapidly. It sorts out apparently well persons who probably have an infection or disease from those who probably do not (ECDC/EMCDDA, 2023). |  |
| **Stages of care cascade** | Steps required to progress from the diagnosis of an infection or disease to treatment to viral suppression or disease cure (Harrison et al., 2019; Zhou et al., 2016). In the context of this study, the stages cover: 1) community-based testing, 2) linkage to care and/or 3) adherence to treatment for hepatitis B and C, HIV and tuberculosis. |  |
| **Telemedicine** | The use of telecommunication technologies for delivery of health services where clients/patients and healthcare providers are separated by a distance (ECDC/EMCDDA, 2023). |  |

**References**

Boon, H. S., Mior, S. A., Barnsley, J., Ashbury, F. D., & Haig, R. (2009). The Difference Between Integration and Collaboration in Patient Care: Results From Key Informant Interviews Working in Multiprofessional Health Care Teams. *Journal of Manipulative & Physiological Therapeutics, 32*(9), 715-722. doi: 10.1016/j.jmpt.2009.10.005

Cullum, N., Spilsbury, K., & Richardson, G. (2005). Nurse led care. *BMJ, 330*(7493), 682. doi: 10.1136/bmj.330.7493.682

ECDC. (2022). Models of good practice for community-based testing, linkage to care and adherence to treatment for hepatitis B and C, HIV and tuberculosis and for health promotion interventions to prevent infections among people who inject drugs. Stockholm: ECDC

ECDC/EMCDDA. (2023). Prevention and control of infectious diseases among people who inject drugs: first update 2023. Stockholm: ECDC

EMCDDA. (2020). European responses to the needs of people who experience homelessness and use drugs. Background paper commissioned by the EMCDDA for Health and social responses to drug problems: a European guide. Publications Office of the European Union, Luxembourg: EMCDDA

Feder, M., Klinedinst, S., & Yee, K. A. (2018). Strategies and Approaches for Developing and Implementing Hepatitis C Medical Case Management. Findings from the Literature Review. Available from: <https://cardeaservices.org/wp-content/uploads/2021/09/HEPLitReview.pdf> [Accessed 4 October 2022]: Cardea

Goodwin, N. (2016). Understanding Integrated Care. *International journal of integrated care, 16*(4), 6. doi: <https://doi.org/10.5334/ijic.2530>

Harrison, G. I., Murray, K., Gore, R., Lee, P., Sreedharan, A., Richardson, P., Hughes, A. J., Wiselka, M., Gelson, W., Unitt, E., Ratcliff, K., Orton, A., Trinder, K., Simpson, C., Ryder, S. D., Oelbaum, S., Foster, G. R., Christian, A., Smith, S., Thomson, B. J., Reynolds, R., Harris, M., Hickman, M., & Irving, W. L. (2019). The Hepatitis C Awareness Through to Treatment (HepCATT) study: improving the cascade of care for hepatitis C virus-infected people who inject drugs in England. *Addiction, 114*(6), 1113-1122. doi: <https://doi.org/10.1111/add.14569>

Scottish Executive. (2002). Integrated care for drug users: principles and practices. Edinburgh: Substance Misuse Division, Scottish Executive

UNODC. (2012). Outreach for Injecting Drug Users. Standard Operating Procedure. Vienna: United Nations Office on Drugs and Crime

Vanderplasschen, W., Rapp, R. C., De Maeyer, J., & Van Den Noortgate, W. (2019). A Meta-Analysis of the Efficacy of Case Management for Substance Use Disorders: A Recovery Perspective. *Frontiers in Psychiatry, 10*.

WHO. (2018). Guidelines for the care and treatment of persons diagnosed with chronic hepatitis C virus infection. Geneva. Accessed from <https://www.who.int/publications/i/item/9789241550345> . WHO

Zhou, K., Fitzpatrick, T., Walsh, N., Kim, J. Y., Chou, R., Lackey, M., Scott, J., Lo, Y.-R., & Tucker, J. D. (2016). Interventions to optimise the care continuum for chronic viral hepatitis: a systematic review and meta-analyses. *The Lancet Infectious Diseases, 16*(12), 1409-1422. doi: 10.1016/s1473-3099(16)30208-0
