# Supplementary material for: Models of good practice to enhance infectious disease care cascades among people who inject drugs: a qualitative study of interventions implemented in European settings
Source: BMC Health Serv Res. 2023 Dec 4;23:1352. doi: 10.1186/s12913-023-10412-y (PMC10696743; doi:10.1186/s12913-023-10412-y)
Supplement: Supplementary file 2 — Supplementary Material 2: Table S2.1 Models of good practice: Online reporting form for interventions to increase stages of care cascade among PWID (A-C). Table S2.2 Models of good practice: Assessment form for interventions to increase stages of care cascade (A-C). [file 12913_2023_10412_MOESM2_ESM.docx]

**Supplementary material 2**

**Table S2.1 Models of good practice: Online reporting form for interventions to increase stages of care cascade among PWID (A-C)**

| **Stage of care cascade** | **Link to online reporting form** | |
| --- | --- | --- |
| **Community-based testing (A)** | [**https://ec.europa.eu/eusurvey/runner/SFMOP2020_A**](https://ec.europa.eu/eusurvey/runner/SFMOP2020_A) |  |
| **Linkage to care (B)** | [**https://ec.europa.eu/eusurvey/runner/SFMOP2020_B**](https://ec.europa.eu/eusurvey/runner/SFMOP2020_B) |  |
| **Adherence to treatment (C)** | [**https://ec.europa.eu/eusurvey/runner/SFMOP2020_C**](https://ec.europa.eu/eusurvey/runner/SFMOP2020_C) |  |

**Table S2.2 Models of good practice: Assessment form for interventions to increase stages of care cascade (A-C)**

For inclusion and core criteria a score of 1 (applicable) or 0 (not applicable) has been given. For each qualifier criteria a score has been given on a scale from 0 to 5, where 0 = not applicable and 5 = fully **a**

| 1. **Interventions to improve community-based testing for PWID** 2. **Interventions to increase linkage to care for PWID** 3. **Interventions to increase adherence to treatment of infections among PWID** | | | |
| --- | --- | --- | --- |
| **PROJECT DETAILS** |  | |  |
| Name of project |  |  | |
| Author |  |  | |
| Affiliation(s) |  |  | |
| Contact |  |  | |
| **INCLUSION CRITERIA** |  | | *Indicate 1/0* |
| Country | Does the MoGP refer to EU/EEA member state, the UK or a country in the European Neighbouring Policy (ENP) area or the Western Balkans? |  | |
| Timeframe | Does the MoGP meets the defined timeframe (2011 until now)? |  | |
| Intervention field | Does the MoGP apply to one of the intervention areas/types (A-C)? |  | |
| Infection(s) addressed | Does the MoGP refer to one of the following infections: HCV, HBV, HIV, TB? |  | |
| PWID (sub-)population | Does the MoGP explicitly refer to PWID (sub-)populations? |  | |
|  | **Total score to meet criteria: minimum 5 out of 5** |  | |
| **CORE CRITERIA** |  | | *Indicate 1/0* |
| Effectiveness | Is a description of the rational/background/context provided? |  | |
|  | Are effectiveness indicators clearly defined? |  | |
|  | Are effectiveness indicators reported? |  | |
|  | Does the intervention show an impact? |  | |
|  | Is/has monitoring and evaluation of activities performed? |  | |
|  | Are publications of results available? (e.g. peer-reviewed literature, grey literature, annual reports) |  | |
|  | **Total score to meet criteria: minimum 5 out of 6** |  | |
| **SPECIFIER CRITERIA** |  | | *To be listed* |
| Setting | Is the project/programme linked to specific settings or epidemiological context? |  | |
| Target population | Is the project/programme referring to specific sub-populations of PWID? |  | |
| **QUALIFIER CRITERIA** |  | | *Score 0 – 5* |
| Transferability^1^ | Is the model transferable to other settings? |  | |
| Sustainability^2^ | Is the model sustainable? |  | |
| Participation^3^ | Do you consider participatory aspects as adequate? |  | |
| Inter-sectoral collaboration^4^ | Do you consider involved system partners as relevant? |  | |
|  | **Total score to meet criteria: minimum 12 out of 20** |  | |
| **Expert's comments** *(please sum up your opinions, comments, views and state the final appraisal outcome)* | | | |

^1^Transferability - the evaluator may consider legal aspects, cost, coverage etc;

^2^Sustainability - defined as funding, type of project, policy support/linkage;

^3^Participation - includes involvement of peers and other population groups;

^4^Inter-sectoral collaboration - includes the involvement of key partners and sectors within the development and/or delivery of the intervention.
